# Clinical study of ganshuang granule combined with tenofovir in the treatment of chronic hepatitis B complicated with nonalcoholic fatty liver disease

**DOI:** 10.3389/fphar.2022.1032789

**Published:** 2022-12-14

**Authors:** Changtian Su, Qin Yang

**Affiliations:** Department of Liver Disease, Sichuan Leshan Traditional Chinese Medicine Hospital, Leshan, China

**Keywords:** ganshuang granule, chronic hepatitis B, non-alcoholic fatty liver disease, antiviral therapy, clinical trial

## Abstract

**Objective:** This study aims to investigate the clinical efficacy of Ganshuang granules combined with tenofovir, an antiviral drug, in the treatment of chronic hepatitis B complicated with nonalcoholic fatty liver disease.

**Methods:** A total of 92 patients with chronic hepatitis B combined with non-alcoholic fatty liver who were treated in our Hospital from January 2020 to December 2021 were included as the research objects. According to the method of random number table, the patients were divided into the control group (*n* = 42) and the treatment group (*n* = 50). The control group was treated with silibinin meglumine tablets and tenofovir, while the treatment group was treated with Ganshuang granules combined with silybin meglumine tablets and tenofovir. Before and after treatment, liver function index, liver hardness measurement (LSM), controlled attenuation parameter (CAP), HBV-DNA serum load and body mass index (BMI) were observed.

**Results:** Compared with the baseline, ALT, AST and GGT were significantly improved in both groups after treatment (*p* < 0.05), while TBIL indexes were not significantly different before and after treatment (*p* > 0.05). Patients in the treatment group had significantly lower ALT and AST index values than the control group at 12 and 24 weeks of treatment (*p* < 0.05). At 12 and 24 weeks of treatment, the fat attenuation parameters of the two groups were significantly decreased compared with those before treatment, and the difference was statistically significant (*p* < 0.05). The fat attenuation parameters in the treatment group were significantly lower than those in the control group at 12 and 24 weeks after treatment (*p* < 0.05).

**Conclusion:** The effect of Ganshuang granule combined with antiviral drugs in the treatment of chronic hepatitis B complicated with non-alcoholic fatty liver is significantly better than that of antiviral drugs alone, which is worthy of clinical recommendation.

**Systematic Review Registration:** https://register.clinicaltrials.gov, identifier NCT05523648.

## 1 Introduction

Chronic hepatitis B (CHB) is a chronic infectious disease caused by hepatitis B virus (HBV) infection with an inflammatory response that results in severely impairment of liver function. Hepatitis B virus infection is a global health problem (Liu et al., 2019a). It is reported that about 650,000 people die each year from liver failure, cirrhosis and liver cancer associated with HBV infection (Lozano et al., 2012). Current treatments for CHB include the immunomodulatory interferon and nucleoside (acid) analogs that inhibit reverse transcriptase (Ying et al., 2018). Common antiviral drugs include lamivudine, adefovir, telbivudine, and tenofovir.

As a metabolic stress-induced liver injury, Non-alcoholic fatty liver disease (NAFLD) not only directly leads to liver damage or death, but also increases the risk of metabolic syndrome (Godoy-Matos et al., 2020), cardiovascular disease (Miptah et al., 2020), and type 2 diabetes (Marušić et al., 2021). In recent years, NAFLD has become the most common chronic liver disease, with a current global prevalence of 25.24% (Martín-Mateos and Albillos, 2021). Common clinical antioxidants such as silymarin can effectively relieve liver oxidative stress and improve liver fibrosis in NAFLD patients (Liang et al., 2018). However, these anti-fibrotic drugs still lack sufficient evidence.

With the rising prevalence of NAFLD, the number of chronic hepatitis B patients with non-alcoholic fatty liver disease has also increased. It is estimated that 25%–30% of CHB patients also have NAFLD (Sharif et al., 2019). NAFLD and CHB synergistically exacerbate liver injury and increase the risk of cirrhosis and HCC (Chan et al., 2017; Lee et al., 2019; Choi et al., 2020; Kim et al., 2021). In a cross-sectional study of patients with CHB, more than 10% of hepatic steatosis was independently associated with severe liver fibrosis (Petta et al., 2011). In addition, patients with chronic hepatitis B and nonalcoholic fatty liver disease have a worse prognosis on antiviral therapy than patients with CHB alone (Cai et al., 2018; Kim et al., 2021). Therefore, it is of extraordinary significance to seek new and efficient treatment options for CHB patients complicated with NAFLD.

Ganshuang Granules is a prescription drug for the treatment of liver fibrosis. Ganshuang granules are composed of traditional Chinese medicines such as Codonopsis pilosula, Radix Bupleuri, Radix Paeoniae Alba, Radix Angelicae Sinensis, Poria, Fructus aurantii, Atractylodes, Polygonum cuspidatum, Dandelion, Prunella, Salvia, Peach Kernel, Turtle shell. Ganshuang granule, as traditional Chinese medicine, has unique advantages in the treatment of liver fibrosis due to its multi-target pharmacological properties (Zeng et al., 2020; Zhao et al., 2021). Comparing with the target therapy of Western medicine, traditional Chinese medicine may have certain advantages in the treatment of combined diseases. For example, in this study, two antiviral drugs were used to treat chronic hepatitis B and nonalcoholic fatty liver disease, and Ganshuang granules is likely to have therapeutic effects on both diseases.

## 2 Materials and methods

### 2.1 General information

This study was registered at https://register.clinicaltrials.gov (registration number: NCT05523648).

A total of 92 patients with chronic hepatitis B combined with nonalcoholic fatty liver who were treated in Leshan Traditional Chinese Medicine Hospital from January 2020 to December 2021 were included as the research objects, and all patients did not receive antiviral therapy. According to the method of random number table, the patients were divided into control group and treatment group. Randomization and subsequent data statistics were performed by physicians and research assistants in our department. This study was performed in agreement with the declaration of Helsinki, and has been approved by the ethics committee of our hospital (ethics number: 202004). All patients provided informed consent and signed informed consent forms.

### 2.2 Inclusion and exclusion criteria

Inclusion criteria: ① Patients who meet the diagnostic criteria for chronic viral infection type B, are HBV-DNA positive and are not treated with antiviral drugs. ② The patient met the definition of NAFLD and had an ultrasound and imaging diagnosis of fatty liver; ③ The patient was 20–55 years of age. ④ No history of excessive alcohol consumption.

Exclusion criteria: ① chronic hepatitis not caused by hepatitis B virus infection. ② those who have taken fatty liver treatment drugs, hormones or anti-psychotic depressants within the last 30d. ③ patients with liver cirrhosis, liver failure and liver cancer. ④ Patients with heart disease, diabetes, hypertension, autoimmune diseases. ⑤ Patients who refuse to sign informed consent.

### 2.3 Diagnostic criteria

Diagnostic criteria of CHB disease: According to the criteria of AASLD guidelines for treatment of chronic hepatitis B (Infectious Diseases Branch of Chinese Medical Association, 2020), there have been hepatitis B virus infection, HBsAg positive or HBVDNA positive for more than 6 months.

Diagnostic criteria of NAFLD: NAFLD was defined according to the guidelines for the diagnosis and treatment of nonalcoholic fatty liver disease (Endocrinology Branch of Chinese Medical Association, 2018). Pathological or imaging examinations were performed to detect the presence of diffuse steatosis in the liver. Fasting B-ultrasound examinations showed enhanced near-field echoes and weakened far-field echoes of the liver, and the intrahepatic duct structure was unclear. In addition, other causes that can lead to fatty liver, such as ethanol (alcohol) abuse, are excluded. For the evaluation of hepatitis B combined with NAFLD, the liver Transient Elastography (TE) technique was used for examination (Zhang et al., 2019). Controlled attenuation parameter (CAP) < 240 db/m, and liver stiffness values <7.3 kpa are normal values.

### 2.4 Treatment methods

92 patients included in the study were randomly divided into two groups, 42 patients in the control group and 50 patients in the treatment group. Both groups of patients received conventional basic treatment such as liver protection. Moreover, in both groups, patients were asked to change their diet structure, focusing on low fat and low sugar, and were instructed to perform appropriate aerobic exercise.

The control group was treated with silybin meglumine tablets (Jiangsu Zhongxing Pharmaceutical Co., Ltd.; National medicine permission number: H32026233; Production batch: 200304) and tenofovir (QILU Pharmaceutical Co., Ltd.; National medicine permission number: H20173185; Production batch: 1L0694DF6). The use of silybin meglumine tablets was 0.2g/time, 3 times/d for 24 weeks; the use of tenofovir was 300 mg/d, 1 time/d for 24 weeks.

The treatment group was treated with Ganshuang granules (Baoding Tianhao Pharmaceutical Co., Ltd.; National medicine permission number: Z20027671; Production batch: 200326) combined with silybin meglumine tablets and tenofovir. silybin meglumine tablets and tenofovir were used in the same way as in the control group, and Ganshuang granules were used 3 g/time, 3 times/d, and the course of treatment was 24 weeks.

### 2.5 Outcome indicators

Before treatment and at 12 and 24 weeks of treatment, liver function indicators, Liver Stiffness Measurement (LSM), Controlled Attenuation Parameter (CAP), HBV-DNA and Body mass index (BMI) were compared between the two groups. Liver function indicators include: Alanine aminotransferase (ALT), Aspartate aminotransferase (AST), serum total bilirubin (TBIL), and glutamyl transpeptidase (GGT). Body mass index (BMI) [BMI = weight (Kg)/height]. Unit is kg/m2.

### 2.6 Statistical analysis

All data were analyzed by SPSS21.0. Continuous variables were described by means ± standard deviation (means ± SD) if they were normally distributed. Categorical variables are expressed as counts (percentage). Continuous variables were described by means ± standard deviation (range) if they were normally distributed. Categorical variables are expressed as counts (percentage). Variables were compared between the two groups using univariate analysis. Continuous variables were compared using the Student’s *t*-test or the Mann-Whitney U-test, depending on their distribution. Categorical variables were compared using the chi-squared test or Fisher’s exact test. *p* < 0.05 indicates that the difference is statistically significant.

## 3 Results

### 3.1 Baseline characteristics of patients in both groups

Of the 92 patients included in the study, no patients discontinued treatment due to adverse events or lack of efficacy. The baseline characteristics of the patients in both groups were shown in Table 1. Of the 42 patients in the control group, 26 (61.9%) were males and 16 (38.1%) were females, with a mean age of 38.98 ± 10.46 years in the control group. Of the 50 patients in the treatment group, 37 (74%) were males and 13 (26%) were females, with a mean age of 37.30 ± 9.65 years in the treatment group. There was no significant difference in baseline characteristics of age, gender structure and BMI between the two groups of patients (*p* > 0.05), which indicated that the two groups of patients were comparable.

**TABLE 1:** Baseline characteristics of patients.

		Control group (*n* = 42)	Treatment group (*n* = 50)	χ2/t	*p* value
Gender	Age (years)	38.98 ± 10.46	37.30 ± 9.65	0.798	0.427
	Male	26 (61.9%)	37 (74%)	1.547	0.214
	Female	16 (38.1%)	13 (26%)		
	BMI (kg/m2)	24.98 ± 1.60	24.82 ± 1.90	0.421	0.675

### 3.2 Recovery of liver function in the two groups before and after treatment

The improvement of each index of liver function before and after treatment in the two groups was shown in Table 2. After 12 weeks and 24 weeks of treatment, there was significant difference in all indexes of liver function in the same group (*p* < 0.05). Compared with the baseline, ALT, AST and GGT were significantly improved in the two groups after 12 and 24 weeks of treatment (*p* < 0.05), and there was no significant difference in TBIL before and after treatment (*p* > 0.05). There was no significant difference in ALT index between the treatment group and the control group before treatment (*p* > 0.05). After 12 and 24 weeks of treatment, the ALT index value of the treatment group was significantly lower than that of the control group (*p* < 0.05). Although the AST index value of the treatment group was significantly higher than that of the control group before treatment (*p* < 0.05), after 12 weeks and 24 weeks of treatment, the AST index value of the treatment group was lower than that of the control group, and the difference was statistically significant (*p* < 0.05).

**TABLE 2:** Improvement of liver function at different time points before and after treatment.

		TBIL (umol/L)	ALT (U/L)	AST (U/L)	GGT (U/L)
Control group (*n* = 42)	Before	18.29 ± 4.85	98.72 ± 90.92	58.86 ± 19.58	83.95 ± 36.38
	After 12 weeks	18.79 ± 3.82	62.50 ± 51.39#	45.98 ± 13.84#	56.53 ± 17.94#
	After 24 weeks	18.75 ± 4.20	46.76 ± 18.15#	41.19 ± 14.60#	51.28 ± 15.80#
Treatment group (*n* = 50)	Before	17.45 ± 2.76	95.40 ± 46.73	80.46 ± 46.21*	90.16 ± 41.05
	After 12 weeks	16.75 ± 2.04*	51.32 ± 23.91#*	43.92 ± 14.86#*	64.94 ± 27.68#*
	After 24 weeks	17.68 ± 8.59*	38.28 ± 9.20#*	35.07 ± 8.00#*	52.19 ± 16.36#

Note: Compared with the baseline, #*p* < 0.05; compared with the control group, *p < 0.05. TBIL, serum total bilirubin; ALT, alanine aminotransferase; AST, Aspartate aminotransferase; GGT, glutamyltranspeptidase.

### 3.3 Comparison of HBV-DNA serum load between two groups of patients

To verify the effect of the drug on the clearance of hepatitis B virus, the number of patients with HBV-DNA serum load <500 IU/ml before and after treatment was counted in this study and the results are shown in Table 3. Before treatment, the HBV-DNA serum load of all patients was greater than 500 IU/ml. After treatment, the number of patients with HBV-DNA serum load <500 IU/ml in both groups increased, indicating that drug treatment is effective in the clearance of hepatitis B virus. At 12 weeks of treatment, the proportion of patients with HBV-DNA serum load <500 IU/ml in the treatment group was significantly higher than that in the control group, and the difference was statistically significant (*p* < 0.05). At 12 weeks of treatment, the proportion of patients with HBV-DNA serum load <500 IU/ml in the treatment group was significantly higher than that in the control group, and the difference was statistically significant (*p* < 0.05). Similarly, at 24 weeks of treatment, the number of patients with HBV-DNA serum load <500 IU/ml in the control group was 31 (73.8%), and the number of patients with HBV-DNA serum load <500 IU/ml in the treatment group was 49, accounting for up to 98%. The number of patients with HBV-DNA serum load <500 IU/ml was significantly higher in the treatment group than in the control group (*p* < 0.05), implying that the combination of liver tonics pellets with antiviral drug therapy was more effective in clearing hepatitis B virus than antiviral drug therapy alone.

**TABLE 3:** Statistics on the number of patients with HBVDNA serum load <500 IU/ml before and after treatment.

	Before treatment	12 weeks after treatment	24 weeks after treatment
Control group (*n* = 42)	0	26 (61.9%)	31 (73.8%)
Treatment group (*n* = 50)	0	43 (86%)	49 (98%)
χ2	—	7.07	11.78
*p* value	—	0.008	0.001

### 3.4 Improvement of liver stiffness in two groups of patients before and after treatment

Patients with CHB combined with NAFLD are often associated with severe liver injury and cirrhosis. In our study, liver sclerosis index was detected using LSM to compare the improvement of liver stiffness before and after treatment in the two groups, and the results are presented in Table 4. The results showed that the liver cirrhosis index in the control group at 12 and 24 weeks after treatment had no significant change compared with the baseline, and the difference was not statistically significant (*p* > 0.05). At 12 and 24 weeks of treatment, the liver cirrhosis index in the treatment group decreased significantly compared with the baseline, and the difference was statistically significant (*p* < 0.05). This indicates that the intervention of Ganshuang Granules may play a better role in improving liver stiffness.

**TABLE 4:** Liver stiffness values at different time points before and after treatment (kpa).

	Before treatment	12 weeks after treatment	24 weeks after treatment
Control group (*n* = 42)	10.99 ± 3.27	10.76 ± 3.60	10.47 ± 3.69
Treatment group (*n* = 50)	12.93 ± 4.143*	11.26 ± 4.07#*	10.05 ± 3.96#

Note: Compared with the baseline, #*p* < 0.05; compared with the control group, *p < 0.05.

### 3.5 Comparison of the improvement of BMI and fat attenuation in the two groups before and after treatment

After antiviral and combined drug interventions, the LSM was used to detect and compare fat attenuation parameters in this study (Table 5). The results showed that at 12 and 24 weeks of treatment, the fat attenuation parameters of the two groups of patients were significantly decreased compared with those before treatment, and the difference was statistically significant (*p* < 0.05). The fat attenuation parameters of the treatment group were significantly lower than those of the control group at 12 and 24 weeks of treatment, and the difference was statistically significant (*p* < 0.05).

**TABLE 5:** Comparison of fat attenuation parameters at different time points before and after treatment in the two groups of patients (db/m).

	Before treatment	12 weeks after treatment	24 weeks after treatment
Control group (*n* = 42)	271.05 ± 15.78	266.22 ± 15.56#	259.20 ± 15.95#
Treatment group (*n* = 50)	271.63 ± 17.74	260.88 ± 14.16#*	252.62 ± 12.81#*

Note: Compared with the baseline, #*p* < 0.05; compared with the control group, *p < 0.05.

## 4 Discussion

Chronic hepatitis B (CHB) and non-alcoholic fatty liver disease (NAFLD) are chronic liver diseases with high prevalence worldwide (Schweitzer et al., 2015; Younossi et al., 2016). Data from Peng et al. (2008) showed that the incidence of hepatic steatosis was higher in patients with chronic hepatitis B than in the general population. Paradoxically, several other studies have shown a reduced risk of NAFLD development in patients with chronic hepatitis B (Cheng et al., 2013; Liu et al., 2013; Huang et al., 2016). It can be seen that the relationship between CHB and NAFLD is still highly controversial (Lozano et al., 2012). However, it is certain that as the incidence of NAFLD patients increases year by year, the incidence of CHB combined with NAFLD follows a rising trend. In addition, NAFLD has also been shown to significantly increase the risk and mortality of liver cancer in CHB patients (Choi et al., 2020). There is great value in the clinical development and evaluation of highly effective treatments for CHB combined with NAFLD.

Previous studies have shown that persistent HBV replication is an independent risk factor for the progression of chronic hepatitis B to cirrhosis and hepatocellular carcinoma (Chen et al., 2006). Reduction of HBV-DNA concentration through long-term antiviral drug therapy reduces the risk of patients developing cirrhosis or liver cancer (Lim et al., 2014). Common antiviral drugs are immunomodulators or nucleoside (acid) analogs (Miptah et al., 2020). Tenofovir disoproxil, a guanosine analog, is recommended as a better antiviral drug for the treatment of CHB (Choi et al., 2019). The primary mechanism of antiviral therapy with nucleoside (acid) analogs is inhibition of viral reverse transcription, which also leads to limitations in their effectiveness in clearing HBsAg. In addition, patients with CHB need to take tenofovir for a long time, which can lead to renal tubular damage and hypophosphatemia (Han et al., 2017). For patients with CHB combined with NAFLD, the bioavailability of antiviral drugs such as tenofovir is reduced due to the accumulation of cellular fat, which reduces the contact area between hepatocytes and therapeutic drugs (Lozano et al., 2012). Therefore, the use of antiviral drugs combined with hepatoprotective drugs shows good advantages in the treatment of CHB patients with NAFLD. Silybin, as a natural hepatoprotective agent, can inhibit intrahepatic fat accumulation by promoting the transcription of Adipose triglyceride lipase (ATGL) gene (Yao et al., 2013). The study by Lv showed that silibinin capsules combined with lifestyle modification is effective in improving hepatic steatosis in patients with chronic hepatitis B (Lv et al., 2021). In our study, the control group was treated with silybin meglumine tablets combined with tenofovir. After 12 and 24 weeks of treatment, the liver function of patients with CHB combined with NAFLD was significantly recovered, the proportion of patients with HBV-DNA serum load <500IU/ml was increased, and liver steatosis was significantly improved. Silibinin has antioxidant, anti-inflammatory, and anti-fibrotic properties that may relieve fatty liver damage (Loguercio and Festi, 2011). Tenofovir exerts an antiviral biological function to inhibit the reverse transcription of HBV and suppress chronic hepatitis B.

Patients with CHB combined with NAFLD require treatment to preserve the liver and improve fibrosis and hepatic steatosis. Traditional Chinese medicine has decades of historical experience in the treatment of chronic hepatitis B liver fibrosis (Song et al., 2019). Ganshuang granules is a prescription drug for the treatment of liver fibrosis. In China, Ganshuang granule combined with antiviral drugs is suitable for the treatment of liver fibrosis in patients with chronic hepatitis B (Zeng et al., 2020). Recent studies have shown that Ganshuang granules can promote the recovery of liver function in mice by inhibiting the differentiation of Treg cells (Liu et al., 2019b). Lu et al. (2021) found that patients with fibrosis and cirrhosis caused by chronic hepatitis B showed greater improvement in liver tissue in a shorter period of time after treatment with entecavir plus liver tonics pellets compared to the entecavir plus placebo group. Similarly, in our study, the treatment group was treated with Ganshuang granules combined with antiviral drugs to treat CHB patients with NAFLD. After 12 and 24 weeks of treatment, patients in the treatment group had greater recovery of liver function and more significant decreases in cirrhosis index and fat attenuation values compared with the control group (*p* < 0.05). In addition, in our study, after 12 weeks and 24 weeks of treatment, the proportion of patients with HBV-DNA serum load <500 IU/ml in the treatment group was significantly higher than that in the control group (*p* < 0.05). All these indicate that when Ganshuang granules combined with antiviral drugs in the treatment of CHB patients with NAFLD, the clearance effect of hepatitis B virus is better than that of antiviral drugs alone. This may also be due to the fact that Ganshuang granules inhibit the accumulation of cell fat, thereby increasing the contact area of antiviral drugs with cells (Shi et al., 2017).

The results of this study strongly suggest that Ganshuang granules combined with antiviral drugs in the treatment of CHB patients with NAFLD has more advantages in protecting liver and improving clinical biochemical indexes than using antiviral drugs alone. Interestingly, there may be multiple potential mechanisms. Naringin in Ganshuang granules controls the activation of hepatic stellate cells by regulating the target of rapamycin in mammals, and then inhibits liver fibrosis. Ganshuang granules inhibits oxidative stress in liver tissue of patients with CHB and NAFLD may be another reason. The increase of fatty acid content in the liver of NAFLD patients leads to a compensatory increase in mitochondrial β-oxidation function and the production of a large amount of ROS. At the same time, peroxidase in microparticles was upregulated, which enhanced the oxidation of mitochondria, and a large amount of ATP was consumed, resulting in further accumulation of ROS. Large amounts of ROS disrupt the mitochondrial respiratory chain complex and damage the mitochondrial structure, further damaging the hepatocytes and causing necrosis and apoptosis (Engin, 2017). Salvia miltiorrhiza is one of the components of Ganshuang granule. Salvia miltiorrhiza polyphenolic acid isolated and purified from Salvia miltiorrhiza can reduce liver inflammation and oxidative stress in a NAFLD rat model, thereby exerting hepatoprotective effects (Ding et al., 2016). Saikosaponins in Ganshuang granule can increase the activity of antioxidant enzymes in liver tissue of mice, inhibit oxidative stress and reduce lipid deposition in liver (Chang et al., 2021). Although Ganshuang Granules combined with other drugs or used alone can significantly improve liver fibrosis and significantly reduce liver stiffness in patients with CHB and NAFLD, its specific mechanism of action needs more time to be explored.

Our research still has some limitations. First, our study is a single-center study based on a Chinese population, and our results are limited in terms of clinical generalization. Larger sample and multicenter long-term follow-up data are needed to support our conclusions in the future. Secondly, our patient did not have a liver biopsy. Liver biopsy is considered to be the gold standard for the grading and staging of liver inflammation, fibrosis and steatosis. Third, we are unable to assess the effects of other confounding factors related to metabolic parameters on our results, such as eating habits. In addition, the sample size included in this study was small.

Chronic hepatitis B and nonalcoholic fatty liver disease are both chronic progressive diseases, which can show short-term curative effect, but cannot show the late progress of the disease. Therefore, this paper observed the treatment of patients at 12 weeks and 24 weeks after treatment, respectively, as the evaluation index of short-term and long-term curative effect.

## 5 Conclusion

Ganshuang granules combined with antiviral drugs tenofovir and silybin meglumine tablets can obviously promote the recovery of liver function, improve liver cirrhosis and fibrosis, and inhibit liver fat accumulation in the treatment of chronic hepatitis B complicated with nonalcoholic fatty liver disease. The curative effect of Ganshuang granules combined with antiviral drugs tenofovir and silybin meglumine tablets on chronic hepatitis B complicated with nonalcoholic fatty liver disease is obviously better than that of antiviral drugs alone, which is worthy of clinical recommendation.

## Data Availability

The original contributions presented in the study are included in the article/Supplementary Material, further inquiries can be directed to the corresponding author.
